# Efeitos do Treinamento Intervalado de Alta Intensidade sobre a Pressão Arterial Central: Uma Revisão Sistemática e Metanálise

**DOI:** 10.36660/abc.20220398

**Published:** 2023-03-24

**Authors:** Gustavo Henrique de Oliveira, Rogério Toshiro Passos Okawa, Caroline Ferraz Simões, João Carlos Locatelli, Victor Hugo de Souza Mendes, Higor Barbosa Reck, Wendell Arthur Lopes

**Affiliations:** 1 Departamento de Educação Física Centro de Ciências da Saúde Universidade Estadual de Maringá Maringá PR Brasil Departamento de Educação Física, Centro de Ciências da Saúde, Universidade Estadual de Maringá, Maringá, PR – Brasil; 2 GPHARV Universidade Estadual de Maringá Maringá PR Brasil Grupo de Pesquisa em Hipertensão Arterial Sistêmica, Rigidez Arterial e Envelhecimento Vascular (GPHARV), Universidade Estadual de Maringá,Maringá, PR – Brasil; 3 Departamento de Medicina Centro de Ciências da Saúde Universidade Estadual de Maringá Maringá PR Brasil Departamento de Medicina, Centro de Ciências da Saúde, Universidade Estadual de Maringá, Maringá, PR – Brasil; 4 Faculdade de Ciências Humanas University of Western Australia Perth Austrália Faculdade de Ciências Humanas (Ciências do esporte, exercício e saúde), University of Western Australia, Perth, Western Australia – Austrália

**Keywords:** Treinamento Intervalado de Alta Intensidade, Treino Aeróbico, Hemodinâmica, Pressão Sanguínea, Rigidez Arterial

## Abstract

A pressão arterial central (PAc) é considerada um preditor independente de lesão de órgão, eventos cardiovasculares e mortalidade por todas as causas. Evidências mostram que o treino intervalado de alta intensidade (HIIT) é superior ao treino contínuo de intensidade moderada (MICT) na melhoria da aptidão cardiorrespiratória e da função vascular. No entanto, os efeitos dessas modalidades de treino aeróbico sobre a PAc não foram propriamente revisados. Esta metanálise tem como objetivo investigar os efeitos do HIIT versus MICT sobre a PAc.Conduzimos uma metanálise de ensaios controlados randomizados que compararam HIIT versus MICT sobre a PAc. Os desfechos primários foram Pressão Arterial Sistólica (PAS) central (PASc) e Pressão Arterial Diastólica central (PADc). A PAS periférica (PASp), a PAD periférica (PADp), a Velocidade de Onda de Pulso (VOP) e a captação máxima de oxigênio (VO_2max_) foram analisadas como desfechos secundários. A metanálise das diferenças médias (DM) foi conduzida usando modelos de efeitos aleatórios.Nosso estudo incluiu 163 pacientes recrutados em seis ensaios. Encontramos que HIIT foi superior ao MICT em reduzir PASc (DM = -3,12 mmHg, IC95% -4,75 – 1,50, p = 0,0002) e PAS (DM = -2,67 mmHg, IC95% -5,18 – -0,16, p = 0,04) e aumentar VO_2max_ (DM = 2,49 mL/Kg/min, IC95% 1,25 – 3,73, p = 0,001). No entanto, não foram relatadas diferenças quanto à PADc, PAD ou VOP. O HIIT foi superior ao MICT em reduzir PASc, sugerindo seu potencial papel como uma terapia não farmacológica para a pressão arterial elevada.

**Figura Central:**
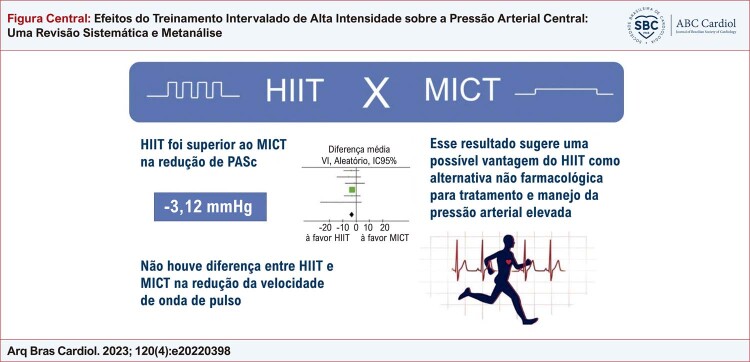
: Efeitos do Treinamento Intervalado de Alta Intensidade sobre a Pressão Arterial Central: Uma Revisão Sistemática e Metanálise

Principais resultados do estudo. HIIT: treino intervalado de alta intensidade; MICT: treino contínuo de intensidade moderada.

## Introdução

As doenças cardiovasculares (DCVs) são a principal causa de doença em todo o mundo, sendo responsáveis por aproximadamente 17,9 milhões de mortes por ano, e um total de 31% de mortalidade por todas as causas.^1^ A pressão arterial (PA) elevada é atualmente o principal fator de risco modificável para DCV e morte prematura.^2^ A pressão arterial sistólica (PAS) elevada (≥140 mmHg) tem sido responsável por 40% das doenças cardíacas, 38% dos acidentes vasculares cerebrais (AVCs) isquêmicos, e 43% das mortes por AVCs hemorrágicos.^3^ No entanto, evidências crescentes sugerem que a pressão arterial central (PAc) é um preditor independente de lesão de órgão, eventos cardiovasculares e mortalidade por todas as causas, uma vez que sua associação com risco cardiovascular é mais forte que a da PA.^4 - 6^ Um estudo recente^7^ mostrou que a PAc foi capaz de predizer o desenvolvimento de hipertensão na população geral.^6^ Além disso, ensaios clínicos demonstraram que drogas anti-hipertensivas podem exercer efeitos divergentes sobre a PAc e a PA.

A PAc pode ser avaliada de maneira não invasiva por vários aparelhos, por análise de ondas de pulso pela tonometria de aplanação utilizando uma função de transferência generalizada, que corrige quanto à amplificação de onda de pressão nos membros superiores.^8^ A análise de onda de pulso representa a forma de onda da pressão aórtica derivada do pulso radial ou carotídeo.^8^ A onda de pulso é composta de uma onda incidente (que se propaga para a frente), formada pela ejeção ventricular esquerda. Quando essa onda incidente alcança as bifurcações ao longo da árvore arterial, ela gera uma onda refletida.^8^ As formas de onda de pressão central são definidas por vários componentes, como a PAS central (PASc), a pressão arterial diastólica central (PADc), e a pressão de pulso central (PPc), as quais são derivadas da função de transferência generalizada e o índice de aumento (AI, *augmentation index* ), definido como a amplitude da onda refletida em termos de PPc, representando a integração da onda de pressão incidente com a onda de pressão refletida.^8^

A atividade física regular é considerada uma abordagem preventiva e um tratamento não farmacológico de primeira linha para hipertensão.^9^ O treino aeróbico (TA) tem sido fortemente recomendado para reduzir a PA.^10 , 11^ Em termos de PAc, em uma metanálise recente, Zhang et al.^12^ observaram uma redução de 5,9 mmHg na PAc após o TA. Esses achados reforçam o potencial do TA em melhorar não só a resistência vascular periférica, como a complacência arterial central, contribuindo para uma redução na PA e na PAc.

O treino intervalado de alta intensidade (HIIT, do inglês *high-intensity interval training* ), parece ser igual ou superior ao treino contínuo de intensidade moderada (MICT, *moderate-intensity continuous training* ) em promover benefícios à saúde, sendo considerado um TA eficiente.^13 - 15^ Ainda, o HIIT apresenta uma taxa de adesão superior e nível de satisfação similar em comparação ao MICT.^16^ Contudo, a comparação entre HIIT e MICT é menos clara em termos de mudanças na PA. Costa et al.^17^ não encontraram diferenças entre HIIT e MICT na redução da PAS ou PAD em indivíduos hipertensos e pré-hipertensos. Por outro lado, Leal et al.^18^ relataram que o HIIT foi superior ao MICT na redução de PAD em indivíduos hipertensos, e Way et al.^19^ observaram que o HIIT foi superior ao MICT em reduzir PAD noturna em adultos.

Com base no exposto, o HIIT surgiu como uma alternativa promissora, uma vez que diretrizes globais de hipertensão recomendam a prática de atividade física regular, incluindo MICT e HIIT, como um componente essencial de mudança de estilo de vida no tratamento de hipertensão.^20^ Contudo, os efeitos do HIIT em comparação ao MICT sobre a PAc ainda não foram revisados adequadamente. Portanto, o objetivo do presente estudo foi revisar os efeitos do HIIT sobre a PAc e compará-lo ao do MICT. Como um desfecho secundário, comparamos a eficácia do HIIT versus HIIT sobre a PA, rigidez arterial e aptidão cardiorrespiratória. Nossa hipótese é que o HIIT seria superior ao MICT em reduzir a PASc.

## Métodos

Esta revisão sistemática e metanálise foi registrada no PROSPERO (CRD42018111573) e conduzida de acordo com as diretrizes PRISMA.^21^

### Estratégia de busca

A busca sistemática por referências foi conduzida em cinco banco de dados eletrônicos (Pubmed/Medline, *Web of Science* , Cochrane, Lilacs e Scielo). Os termos de busca foram definidos previamente e aplicados uniformemente em todos os bancos de dados por dois pesquisadores independentes (GHO e VHSM), a fim de se verificar se o mesmo número de referências era obtido. Os termos usados nas buscas foram os seguintes: ‘central blood pressure’ OR ‘central hemodynamics’ OR ‘aortic systolic blood pressure’ OR ‘aortic blood pressure’ OR ‘central diastolic blood pressure’ OR ‘central systolic blood pressure’ OR ‘arterial stiffness’ OR ‘pulse wave velocity’ OR ‘augmentation index’ AND ‘high-intensity interval training’ OR ‘aerobic interval training’ OR ‘aerobic exercise’ OR ‘moderate-intensity continuous training’ OR ‘HIIT’ OR ‘MICT’ AND ‘randomized controlled trial’ (Tabela suplementar 1). Uma busca por estudos potencialmente elegíveis também foi conduzida na seção de referências. A busca incluiu todas as referências disponíveis do início a 12 de abril de 2022.

### Critérios de elegibilidade

Os critérios de elegibilidade foram estabelecidos segundo a estratégia PICOS – População, Intervenção, Comparação, Desfechos e Delineamento do estudo (do inglês *Population, Intervention, Comparison, Outcomes and Study design* ). Esta revisão incluiu estudos com adultos (≥18 anos de idade) de ambos os gêneros, não atletas e sem restrições em termos de atividade física. A intervenção incluiu estudos usando HIIT conforme definido por Weston et al.,^15^ isto é, um estímulo repetido em intensidade vigorosa (80-100% pico da frequência cardíaca) intercalado com períodos de recuperação (ativa ou passiva). O HIIT foi comparado ao MICT, o qual consistiu em um estímulo contínuo, de intensidade moderada (54-76% do pico da frequência cardíaca ou equivalente). O desfecho primário foi PAc medida antes e após as intervenções de TA, e os desfechos secundários foram pressão arterial, rigidez arterial e aptidão cardiorrespiratória. Somente ensaios controlados randomizados foram considerados nesta revisão.

### Seleção dos estudos

As referências foram sistematizadas com a ajuda de um software específico (Mendeley^®^, Elsevier, Amsterdã, Holanda). Dois pesquisadores (GHO e VHSM) conduziram a busca dos estudos de forma independente. Estudos cujos escopos estavam definitivamente fora do objetivo deste estudo e dados duplicados foram inicialmente excluídos do processo de rastreamento. As referências restantes foram avaliadas por título e resumo, e aqueles ainda considerados elegíveis passaram pela análise do texto completo. Em caso de discordância entre os dois autores, esses tentaram alcançar um consenso explicando seus pontos de vista e, se ainda houvesse discordância, um terceiro autor (JCL) foi consultado para uma decisão final.

Os critérios de exclusão incluíram (1) artigos duplicados; (2) resumos e artigos de congressos; (3) medidas de desfecho sem PAc; (4) delineamento de estudo agudo; (5) outras intervenções de exercício ou de dieta associadas com HIIT ou MICT; (6) relatos incompletos dos dados do estudo.

### Extração dos dados

A extração dos dados qualitativos e quantitativos foi conduzida de modo independente por dois investigadores (GHO e VHSM), e os dados obtidos foram comparados para evitar erro de extração. Os dados foram extraídos utilizando-se uma planilha padronizada. As variáveis demográficas extraídas de cada estudo foram: país; características da amostra; número de indivíduos/idade; técnica de avaliação hemodinâmica; duração/frequência/modo e protocolo de exercício.^21^

### Avaliação do risco de viés

A ferramenta de avaliação de risco Cochrane^®^ (Cochrane collaboration, Oxford, Reino Unido) foi usada para avaliar o risco de viés dos estudos incluídos.^22^ Essa ferramenta é composta por cinco domínios que, em conjunto, abordam os aspectos metodológicos que possam influenciar os resultados de um ensaio. Cada um dos cinco domínios possui questões específicas que permitem cinco respostas (“sim”, “provavelmente sim”, “não”, “provavelmente não”, e “nenhuma informação”). Com base nas respostas, cada domínio era classificado com risco de viés “baixo”, “incerto” ou “alto”, segundo julgamento do autor (GHO), quem avaliou todos os estudos. O principal objetivo desse processo foi avaliar o rigor da metodologia dos estudos e, portanto, isso não foi usado como um critério de exclusão.

### Análise estatística

Os dados foram inseridos manualmente e reunidos na metanálise, por meio do programa Review Manager^®^ versão 5.3 (Colaboração Cochrane). Dados de PASc, PADc, PAS, PAD, velocidade de onda de pulso (VOP) e captação máxima de oxigênio (VO_2max_) foram apresentados como diferença média (DM), com intervalo de confiança de 95% (IC95%). Para os estudos que não apresentaram o desvio padrão (DP) das mudanças nas variáveis, a conversão para DP ou a imputação do DP foi feita pela equação de acordo com o manual Cochrane,^22^ considerando o coeficiente de correlação baseado nos dados apresentados por Oliveira et al.^23^ O modelo de efeitos aleatórios foi usado. Gráficos de floresta ( *forest plots* ) foram criados para quantificar os efeitos de protocolos de HIIT e MICT sobre a PASc e a PADc. A análise de sensibilidade foi conduzida para examinar a magnitude da influência de cada estudo sobre os desfechos. Um nível de significância de p ≤ 0,05 foi adotado. A heterogeneidade dos estudos foi avaliada usando o I2.^22^

## Resultados

### Busca na literatura

**Figura 1:**
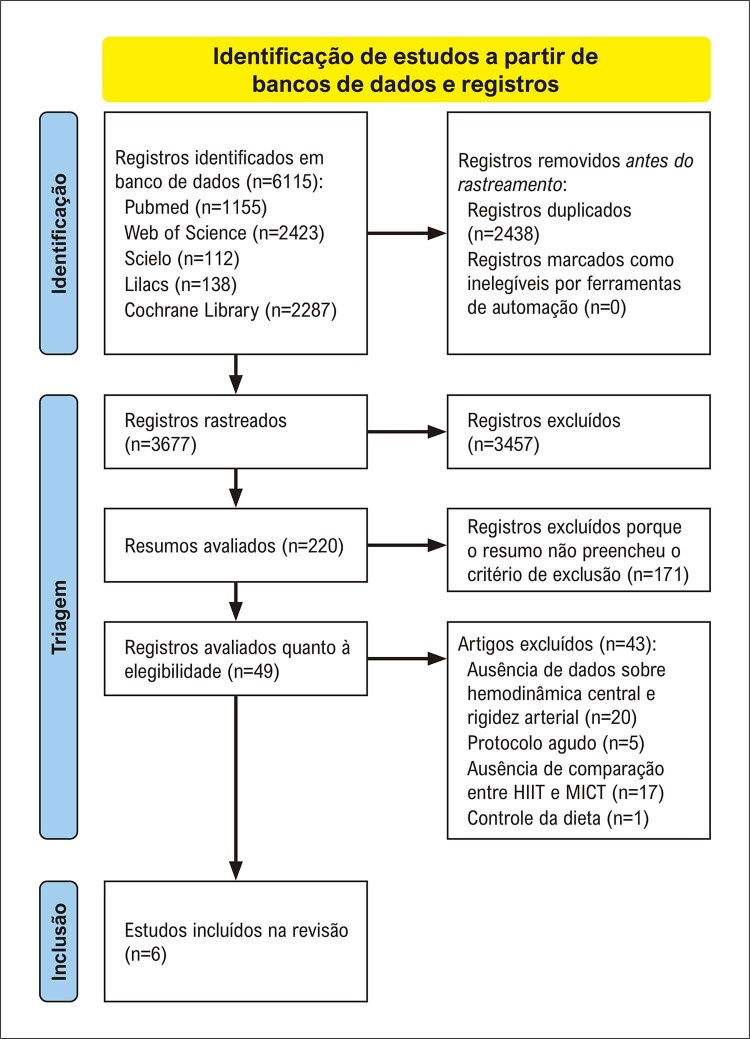
– Fluxograma PRISMA da seleção dos estudos para a revisão sistemática e metanálise.

As buscas iniciais resultaram em 6115 referências. Após remoção das duplicatas, 3677 referências permaneceram para posterior análise dos títulos. Subsequentemente, 3457 referências foram removidas por não se adequarem às questões do PICOS. Após leitura dos resumos, 171 referências foram removidas por não atingirem os critérios de inclusão. Das 49 referências selecionadas para avaliação do texto completo, 43 referências foram excluídas. Seis estudos preencheram os critérios de elegibilidade e foram incluídos na presente revisão sistemática e metanálise ( Figura 1 ).

### Características do estudo

Os estudos incluídos foram publicados entre 2016 e 2020 e conduzidos na Austrália,^24 , 25^ Suíça,^26 , 27^ EUA^28^ e Brasil.^23^ Os estudos foram conduzidos com amostras de 16 a 35 indivíduos, com um total de 163 participantes (70% mulheres). A maioria dos estudos foi conduzido com adultos jovens. Em relação às populações investigadas, os estudos incluíram homens obesos e com sobrepeso,^24^ mulheres obesas,^23^ indivíduos com enxaqueca,^26^ depressão,^27^ idosos sedentários,^28^ e sobreviventes de câncer.^28^ Na avaliação da PAc e rigidez arterial, mais de um método ou técnica foi utilizada, incluindo SphygmoCor^®^ (AtCor Medical, Sidney, Austrália)^23 - 25 , 28^ e o Mobil-O-Graph^®^ (IEM GmbH, Stolberg, Alemanha).^26 , 27^

Em relação à PAc, um estudo relatou reduções significativas na PASc e PADc após HIIT e MICT.^25^ Ainda, reduções importantes na PASc após HIIT e na PADc após MICT foram relatadas em dois estudos.^23 , 24^ Os demais estudos não apresentaram diferenças significativas após os protocolos de HIIT ou MICT.^26 - 28^ Quanto às alterações na VOP, um estudo encontrou uma redução significativa na VOp após MICT.^25 , 28^ Um estudo verificou um aumento na VOP após HIIT.^25^ Os outros três estudos não mostraram mudanças significativas nessa variável.^24 , 26 , 27^

### Descrição dos estudos incluídos

Os programas de TA consistiram em pedalar em um cicloergômetro^24 , 27^ ou em uma bicicleta ergométrica,^23 , 26^ ou correr em uma pista de corrida ou esteira. Um estudo usou tanto a bicicleta como a corrida.^25^ Quanto aos programas de HIIT, três estudos^23 , 27 , 28^ utilizaram o protocolo norueguês tradicional,^26^ que consistiu em quatro sessões de alta intensidade com quatro minutos de duração cada. Dois estudos,^25 , 27^ usaram um protocolo HIIT com sessões de 30 segundos de alta intensidade, variando de sete a 25 estímulos. Um estudo^24^ usou um protocolo HIIT consistindo em 10 sessões de alta intensidade com um minuto cada. Os protocolos de HIIT mantinham uma intensidade entre 85% e 95% da frequência cardíaca máxima. Os protocolos de MICT variaram entre 20 e 47 minutos, com média de duração de 35,4 ± 11 minutos entre os programas, e intensidade entre 55% a 75% da frequência cardíaca máxima. As intervenções duraram entre quatro a 12 semanas, com uma frequência entre duas a quatro vezes por semana. As características gerais dos estudos incluídos estão apresentadas na Tabela 1 .

**Tabela 1:** – Principais características dos estudos incluídos na revisão sistemática e metanálise

Referências	País	Características da amostra	Número de indivíduos/ idade	Técnica de avaliação hemodinâmica	Duração/ Frequência/ Modo	Protocolos de exercício	

						HIIT	MICT
Oliveira et al.^23^	Brasil	Adultos com obesidade	25 mulheres 28±5 anos HIIT:11 MICT:14	SphygmoCor	8 semanas 3 dias/semana Corrida	4x4 min a 85-90% da FC_max_ intercalado por 3 min de recuperação ativa a 65-75% da FC_max_	41 minutos a 65-75% da FC_max_
Clark et al.^24^	Austrália	Adultos com sobrepeso / obesidade	28 adult men 30±6 years HIIT: 16 MICT: 12	SphygmoCor	6 semanas 2 dias/semana Cicloergômetro	10x1 min a 90-100% do W_max_ (aproximadamente ~90% do HR_max_) seguido por 1 min de recuperação ativa a 15% W_max_	30 min a 35-50% W_max_ (65-75% da FC _max_)
Toohey et al.^25^	Austrália	Sobreviventes de câncer	16 mulheres adultas 51±13 anos HIIT: 8 MICT: 8	SphygmoCor	12 semanas 3 dias/semana Corrida na esteira ou cicloergômetro	7x30 segundos a 85% da FC_max_	20 minutos a 55% da FC_max_
Hanssen et al.^26^	Suíça	Pacientes com enxaqueca episódica	25 mulheres adultas 30±10 anos HIIT: 13 MICT: 12	Mobil-o-graph	12 semanas 2 dias/semana Running	4x4 min a 90-95% da FC_max_ intercalado por 3 min de recuperação ativa a 70% da FC_max_	45 min a 70% da FC_max_
Hanssen et al.^27^	Suíça	Pacientes com depressão unipolar	34 adultos (25 mulheres) 38±12 anos HIIT: 19 MICT: 15	Mobil-o-graph	4 semanas 3 dias/semana Cicloergômetro	25x30 segundos a 80% da VO_2max_ seguidos de repouso absoluto por 30 segundos	20 minutos a 60% da VO_2max_
Kim et al.^28^	EUA	Idosos	35 indivíduos (23 mulheres) 64±1 anos HIIT: 17 MICT: 18	SphygmoCor	8 semanas 4 dias/semana Bicicleta ergométrica	4x4 min a 90% da FC_max_ intercalado por 3 min de recuperação ativa a 70% da FC_max_	47 minutos a 70% da FC_max_

HIIT: treino intervalado de alta intensidade; MICT: treino contínuo de intensidade moderada; FC_max_: frequência cardíaca máxima; VO_2max_: captação máxima de oxigênio; W_max_: watts máximo.

### Avaliação do risco de viés

**Figura 2:**
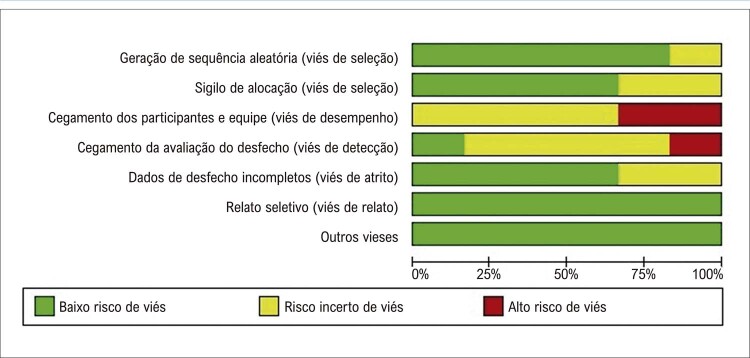
– Avaliação do risco de viés usando a ferramenta de avaliação de Cochrane.

A avaliação do risco de viés dos estudos incluídos mostrou um baixo risco de viés ( Figura 2 ). Somente dois estudos mostraram um alto risco de viés (33,3%), o que indica que os estudos seguiram rigorosamente os procedimentos metodológicos propostos e uma boa qualidade metodológica. Poucas questões relacionadas ao sigilo na alocação e cegamento dos participantes foram observadas em alguns estudos. No entanto, essas questões são limitações típicas em estudos de intervenção com exercício, e não representam, necessariamente, uma baixa qualidade metodológica. Além disso, as limitações observadas foram mencionadas na seção sobre limitações dos respectivos estudos. Vale ressaltar que essa avaliação não foi realizada como um critério de exclusão, sendo usado somente para fins informativos. Os sinais positivos e negativos, e o ponto de interrogação representam, respectivamente, risco baixo, alto e incerto. A análise de sensibilidade com exclusão de um estudo por vez (análise *leave-one-out* ) mostrou que os resultados desta metanálise não foram direcionados por nenhum estudo particular.

### Síntese dos resultados

**Figura 3:**
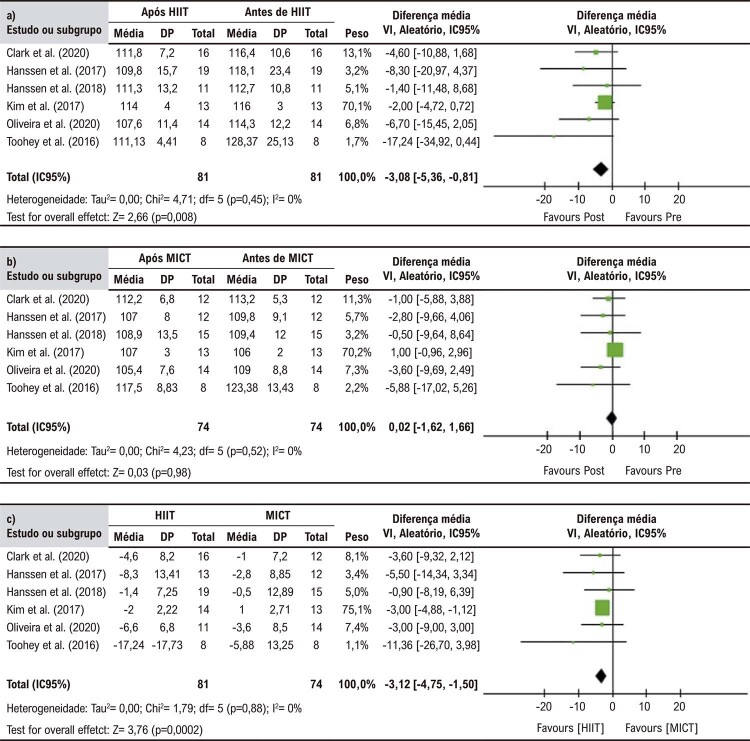
– Forest plot das comparações entre grupos dos efeitos do treino intervalado de alta intensidade (HIIT) versus treino contínuo de intensidade moderada (MICT) sobre a pressão arterial sistólica central; (a) pós-HIIT versus pré-HIIT; (b) pós-MCIT versus pré-MICT; e (c) HIIT versus MICT

O HIIT foi superior ao MICT em reduzir PASc (DM = -3,12 mmHg, IC95%: -4,75 - 1,50, p = 0,0002). Comparações entre os valores basais e pós-exercício mostraram que o HIIT foi capaz de reduzir a PASc significativamente (DM = -3,08 mmHg, IC95%: -5,36 – 0,81, p = 0,008). Em contrapartida, não foram observadas diferenças significativas para MICT DM = 0,02 mmHg, IC95%: -1,62 – 1,66, p = 0,98) ( Figura 3 ).

**Figura 4:**
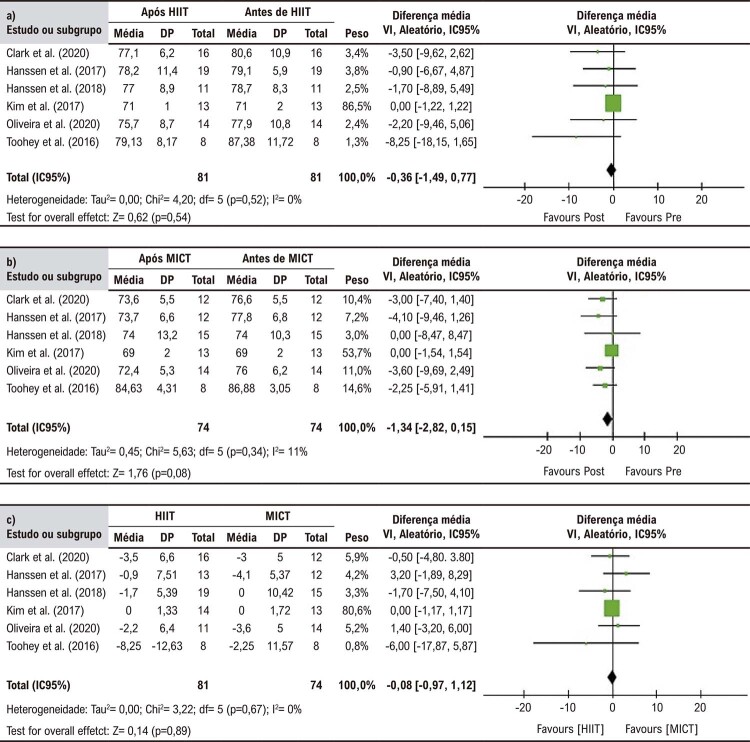
– Forest plot das comparações entre grupos dos efeitos do treino intervalado de alta intensidade (HIIT) versus treino contínuo de intensidade moderada (MICT) sobre a pressão arterial diastólica central; (a) pós-HIIT versus pré-HIIT; (b) pós-MCIT versus pré-MICT; e (c) HIIT versus MICT

A análise agrupada não revelou diferenças significativas entre HIIT e MICT quanto as mudanças na PADc (DM = 0,08 mmHg, IC95%: -0,97 - 1,12, p = 0,89). O mesmo foi observado para HIIT (DM -0,36 mmHg, IC95%: -1,49 – 0.77 mmHg, p = 0,54) e MICT (DM = -1,34 mmHg, IC95%: -2,82 – 0,15, p = 0,08) em comparação a seus respectivos valores basais ( Figura 4 ).

Quanto às variáveis secundárias, a análise agrupada demonstrou que o HIIT foi superior ao MICT na redução da PAS (DM = -2,67 mmHg, IC95% -5,18 – 0,16 mmHg, p = 0,04). Ainda, o HIIT foi superior no aumento do VO_2max_ (DM = 2,49 mL/kg/min, IC95% 1,25 – 3,73, p = 0,001). Contudo, a análise agrupada não mostrou diferenças significativas entre HIIT e MICT para PAD (DM = 0,06 mmHg, IC95%: -1,36 – 1,48], p = 0,94) e VOP (DM = -0,07 m/s, IC95% -1,81 – 1,68, p = 0,94) ( Tabela 2 ).

**Tabela 2:** – Comparação da diferença média e desvio padrão entre o treino intervalado de alta intensidade (HIIT) e o treino contínuo de intensidade moderada sobre a pressão arterial, rigidez arterial e aptidão cardiorrespiratória

Variável	Referências	N	DM	ICI	ICS	p	I^2^	P
**PAS**	23,24,25,26,27,28	154	-2,67	-5,18	-0,16	0,04	0%	0,91
**PAD**	23,24,25,26,27	139	0,06	-1,36	1,48	0,94	0%	0,98
**VOP**	23,24,25,26,27,28	153	-0,07	-1,81	1,68	0,94	0%	0,85
**VO_**2max**_**	24,25,26,27	111	2,49	1,25	3,73	0,001	0%	0,82

PAS: pressão arterial sistólica; PAD: pressão arterial diastólica; VOP: velocidade da onda de pulso; VO_2max_: captação máxima de oxigênio; DM: diferença média; ICI: intervalo de confiança inferior; ICS: intervalo de confiança superior; p: valor p para comparação entre grupos; I^2^: heterogeneidade; P: valor p para heterogeneidade; N: número de participantes.

## Discussão

Este é o primeiro estudo a sistematizar e comparar os efeitos do HIIT versus MICT sobre a PAc em indivíduos sadios e com doença crônica. O principal achado desta metanálise foi a superioridade do HIIT sobre o MICT em reduzir a (DM = -3,12 mmHg, IC95% -4,75 – -1,50, p = 0,0002. Metanálises anteriores mostraram que o HIIT e o MICT são igualmente eficazes em melhorar a PA ambulatorial em indivíduos pré-hipertensos e hipertensos.^17 , 18^ O HIIT e o MICT promoveram uma redução de 5,6 e 3,7 mmHg na PASp e de 4,8 e 2,4mmHg na PAD, respectivamente, em indivíduos hipertensos.^18^ Nossos resultados contribuem ao conhecimento atual em demonstrar que o HIIT parece ser superior ao MICT em reduzir PASc, com uma redução de 3,2mmHg. Contudo, não houve diferenças estatísticas entre HIIT e MICT quanto às alterações na PASc.

Embora a PAp seja amplamente usada na prática clínica, há evidências consistentes de que a PAc seja um preditor independente superior de lesão de órgão e mortalidade cardiovascular em comparação à PAp.^4 , 5 , 7^ Contudo, apesar de evidências corroborando a importância prognóstica da PAc e sua resposta a drogas anti-hipertensivas em comparação à PAp, pouco se sabe sobre o impacto do treinamento com exercício sobre a PAc. Uma metanálise recente conduzida por Zhang et al.^12^ encontrou uma redução de aproximadamente 6mmHg na PASc após o TA. Por outro lado, Evans et al.^29^ não encontraram uma redução significativa na PASc (DM = -3,58 mmHg, IC95% -8,17 – 1,01, p = 0,13) após o treino de resistência isolado ou combinado com TA. A presente metanálise demonstra que o HIIT é superior ao MICT em reduzir PASc, mas não diferente quanto à PADc. Assim, além dos efeitos distintos que algumas drogas anti-hipertensivas exercem sobre a PA dependendo do local analisado, o tipo de TA também pode exercer diferentes efeitos sobe a PA.

Os mecanismos pelos quais o HIIT poderia reduzir a PAc são ainda incertos. Enquanto o HIIT parece ser similar ao MICT em reduzir a rigidez arterial,^19^ ele parece ser superior ao MICT em melhorar a função endotelial.^14 , 30^ Isso contribuiria para a redução da resistência vascular periférica, o que atenuaria o desajuste na impedância entre os vasos centrais e periféricos, diminuindo a velocidade da onda refletida para a aorta, e consequentemente menor amplificação da PASc.

Na segunda análise, encontramos uma superioridade do HIIT em comparação ao MICT em reduzir PAS (DM = -2.67 mmHg, IC95% -5,18 – -0,16, p = 0,04). Esse resultado diverge do relatado em outras metanálises em que não se encontraram diferenças na PAS entre modalidades de TA em pacientes hipertensos e pré-hipertensos.^17 , 18^ Tal discrepância pode estar relacionada à ausência de indivíduos com hipertensão em nosso estudo, o que geraria uma resposta diferente ao TA, particularmente após o HIIT. Considerando o fenômeno de amplificação da onda de pressão,^8 , 31^ seria esperado que mudanças importantes na PAc, promovidas pelo HIIT, seriam transferidas à periferia, resultando em uma redução na PAp. Ainda, o HIIT também foi superior em aumentar a aptidão cardiorrespiratória, corroborando estudos anteriores que compararam essas modalidades de TA sobre o VO_2max_ em diferentes populações.^14 , 15 , 30^ Por outro lado, não encontramos diferenças significativas para VOP entre HIIT e MICT (DM = -0,07 m/s, IC95%: -1,81 – 1,68, *p* = 0,94). Esses achados estão de acordo com os relatados por Way et al.,^15^ que não observaram diferenças significativas entre HIIT e MICT para VOP (DM = 0,004 m/s, IC95% -0,25 – -0,26 m/s, *p* = 0,97). Os principais resultados estão ilustrados na ilustração central.

Este estudo tem algumas limitações que merecem consideração. Primeiro, há uma clara escassez de estudos analisando a PAc, mesmo essa sendo considerada um indicador forte e clinicamente relevante de risco cardiovascular. Uma vez que essa medida não tem sido muito utilizada nos estudos, tivemos que combinar estudos com diferentes populações e condições clínicas na análise agrupada. Ainda, os diferentes métodos para a prescrição de TA, além de diferentes equipamentos usados para a PAc e rigidez arterial podem influenciar as análises. Ainda, apesar de validados, os métodos usados para avaliar PAc utilizam métodos indiretos por oscilometria, o que deve ser interpretado com cuidado. Outra limitação que vale ser mencionada é o fato de que a avaliação do risco de viés foi conduzida por um único pesquisador. Por fim, embora tenhamos rigorosamente seguido as diretrizes PRISMA, algumas referências podem ter sido erroneamente ignoradas.

## Conclusão

Em resumo, o HIIT foi superior ao MICT em reduzir a PASc. No entanto, não houve diferença entre o HIIT e o MICT em relação à PAc ou à VOP. Esse é um achado relevante considerando que a PAc é um forte preditor, clinicamente relevante de eventos cardiovasculares. Estudos futuros são necessários para comparar os efeitos do HIIT e do MICT sobre a PAc em populações específicas, tais como indivíduos pré-hipertensos e hipertensos, os quais são mais expostos a disfunções nos parâmetros hemodinâmicos.

## * Material suplementar

Para tabela suplementar 1, por favor, clique aqui .

Para tabela suplementar 2, por favor, clique aqui .
