# Supplementary material for: Efeitos do Treinamento Intervalado de Alta Intensidade sobre a Pressão Arterial Central: Uma Revisão Sistemática e Metanálise
Source: Arq Bras Cardiol. 2023 Mar 24;120(4):e20220398. [Article in Portuguese] doi: 10.36660/abc.20220398 (PMC10263426; doi:10.36660/abc.20220398)
Supplement: Translation

# Translation: Effects of High-Intensity Interval Training on Central Blood Pressure: A Systematic Review and Meta-Analysis

**Authors:** Gustavo Henrique de Oliveira, Rogério Toshiro Passos Okawa, Caroline Ferraz Simões, João Carlos Locatelli, Victor Hugo de Souza Mendes, Higor Barbosa Reck, Wendell Arthur Lopes

**Affiliations:** 1 Brazil Departamento de Educação Física, Centro de Ciências da Saúde, Universidade Estadual de Maringá, Maringá, PR – Brazil; 2 Brazil Grupo de Pesquisa em Hipertensão Arterial Sistêmica, Rigidez Arterial e Envelhecimento Vascular (GPHARV), Universidade Estadual de Maringá, Maringá, PR – Brazil; 3 Brazil Departamento de Medicina, Centro de Ciências da Saúde, Universidade Estadual de Maringá, Maringá, PR – Brazil; 4 Australia Faculdade de Ciências Humanas (Ciências do esporte, exercício e saúde), University of Western Australia, Perth, Western Australia – Australia

**Keywords:** High-Intensity Interval Training, Endurance Training, Hemodynamics, Blood Pressure, Vascular Stiffness

## Abstract

Central blood pressure (cBP) is considered an independent predictor of organ damage, cardiovascular events and all-cause mortality. Evidence has shown that high intensity interval training (HIIT) is superior to moderate-intensity continuous training (MICT) for improving cardiorespiratory fitness and vascular function. However, the effects of these aerobic training modalities on cBP have not yet been properly reviewed.This meta-analysis aims to investigate to effects of HIIT versus MICT on cBP.We conducted a meta-analysis of randomized controlled trials that compared HIIT versus MICT on cBP. Primary outcomes were measures of central systolic blood pressure (cSBP) and central diastolic blood pressure (cDBP). Peripheral systolic blood pressure (pSBP) and diastolic blood pressure (pDBP), pulse wave velocity (PWV) and maximal oxygen uptake (VO_2max_) were analyzed as second outcomes. Meta-analysis of mean differences (MD) was conducted using the random effects model.Our study included 163 patients enrolled in six trials. We found that HIIT was superior to MICT in reducing the cSBP (MD = -3.12 mmHg, 95% CI: -4.75 to -1.50, p = 0.0002) and SBP (MD = -2.67 mmHg, 95% CI: -5.18 to -0.16, p = 0.04), and increasing VO_2max_(MD = 2.49 mL/kg/min, 95% CI: 1.25 to 3.73, p = 0.001). However, no significant differences were reported for cDBP, DBP and PWV.HIIT was superior to MICT in reducing the cSBP, which suggests its potential role as a non-pharmacological therapy for high blood pressure.

## Introduction

Cardiovascular diseases (CVDs) are the main cause of death worldwide, accounting for approximately 17.9 million deaths per year, and a total of 31% of all-cause mortality.^1^High blood pressure (BP) is nowadays the leading modifiable risk factor for CVD and premature death.^2^Elevated systolic BP (SBP) (≥140 mmHg) has been responsible for 40% of ischemic heart disease, 38% of ischemic stroke and 43% of hemorrhagic stroke deaths.^3^Nevertheless, a growing body of evidence has suggested that central BP (cBP) is an independent predictor of organ damage, cardiovascular events and all-cause mortality, as its association with CVD risk is stronger than BP.^4 - 6^A recent study^7^showed that cBP was able to predict the development of hypertension in the general population.^6^In addition, clinical trials have demonstrated that antihypertensive drugs may exert contrasting effects on cBP and BP.

The cBP can be non-invasively assessed by several devices using pulse wave analysis by applanation tonometry with a generalized transfer function, which corrects for pressure wave amplification in the upper limbs.^8^Pulse wave analysis represents the aortic pressure waveform derived from the radial or carotid pulse.^8^Pulse wave is composed of an incident wave (forward-travelling), formed by the left ventricular ejection. When this incident wave reaches the bifurcations alongside the arterial tree, it generates a reflected wave which travels backwards.^8^Central pressure waveforms are defined by several components, such as central SBP (cSBP), central diastolic blood pressure (cDBP), and central pulse pressure (cPP), which are derived from the generalized transfer function, and the augmentation index, defined as the amplitude of the reflected wave in terms of cPP, representing the integration of the incident and reflected pressure waves.^8^

Regular physical activity is considered a preventive approach and a first-line nonpharmacological treatment for hypertension.^9^Aerobic training (AT) has been strongly recommended to reduce BP.^10 , 11^In terms of cBP, in a recent meta-analysis, Zhang et al.^12^observed a 5.9 mmHg reduction in cSBP after AT. These findings support the potential of AT in improving not only the peripheral vascular resistance but also the central arterial compliance, contributing to a reduction in both BP and cBP.

High-intensity interval training (HIIT) has been reported to be equal or even superior in eliciting health benefits in comparison with moderate-intensity continuous training (MICT), being considered a time-efficient AT.^13 - 15^Also, HIIT has shown a higher adherence rate and similar enjoyment level in comparison to MICT.^16^However, the comparison between HIIT and MICT is less clear in terms of changes in BP. Costa et al.^17^found no differences between HIIT and MICT in reducing SBP or DBP in pre-hypertensive and hypertensive individuals. On the other hand, Leal et al.^18^reported that HIIT was superior to MICT in reducing DBP in hypertensive individuals, and Way et al.^19^found that HIIT was superior to MICT in reducing night-time DBP in adults.

Based on this, HIIT has emerged as a promising alternative, given that current global hypertension guidelines recommend regular physical activity, including MICT or HIIT, as an essential component of lifestyle changing for the treatment of hypertension.^20^However, the effects of HIIT compared to MICT on cBP have not been adequately reviewed yet. Therefore, the aim of the present study was to revise the effects of HIIT versus MICT on cBP. As a secondary outcome, we compared the efficacy of HIIT versus MICT on BP, arterial stiffness and cardiorespiratory fitness. We hypothesized that HIIT would be superior to MICT in reducing cSBP.

## Methods

This systematic review and meta-analysis was conducted according to PRISMA guidelines,^21^and previously registered in PROSPERO (CRD42018111573).

### Search strategy

The systematic search for references was conducted on five electronic databases (Pubmed/Medline, Web of Science, Cochrane, Lilacs and Scielo). The search terms were defined earlier and then uniformly applied in all databases by two independent researchers (GHO and VHSM), aiming to verify whether the same number of references was achieved. The terms used in the searches were the following: ‘central blood pressure’ OR ‘central hemodynamics’ OR ‘aortic systolic blood pressure’ OR ‘aortic blood pressure’ OR ‘central diastolic blood pressure’ OR ‘central systolic blood pressure’ OR ‘arterial stiffness’ OR ‘pulse wave velocity’ OR ‘augmentation index’ AND ‘high-intensity interval training’ OR ‘aerobic interval training’ OR ‘aerobic exercise’ OR ‘moderate-intensity continuous training’ OR ‘HIIT’ OR ‘MICT’ AND ‘randomized controlled trial’ (Supplementary Table 1 ). We also conducted a search in the reference section of potentially eligible studies. The search included all the available references from inception to 12 April 2022.

**Table 1:** – Main characteristics of the studies included in the systematic review and meta-analysis

References	Country	Sample characteristics	Number of subjects/ age	Hemodynamic assessment technique	Length/ Frequency/ Mode	Exercise protocols	
						HIIT	MICT
Clark et al.^23^	Australia	Adults with overweight/ obesity	28 adult men 30±6 years HIIT: 16 MICT: 12	SphygmoCor	6 wks 2 d/wk Cycle ergometer	10x1 min at 90-100% of W_max_(approximately ~90% of HR_max_) followed by 1 min of active recovery at 15% W_max_	30 min at 35-50% W_max_(65-75% of HR_max_)
Hanssen et al.^24^	Switzerland	Patients with episodic migraine	25 adult women 30±10 years HIIT: 13 MICT: 12	Mobil-o-graph	12 wks 2 d/wk Running	4x4 min at 90-95% of HR_max_interspersed by 3 min of active recovery at 70% of HR_max_	45 min at 70% of HR_max_
Hanssen et al.^25^	Switzerland	Patients with unipolar depression	34 adults (25 women) 38±12 years HIIT: 19 MICT: 15	Mobil-o-graph	4 wks 3 d/wk Cycle ergometer	25x30 sec at 80% of VO_2max_followed by 30 sec of absolute rest	20 min at 60% of VO_2max_
Kim et al.^26^	USA	Elderly	35 subjects (23 women) 64±1 years HIIT: 17 MICT: 18	SphygmoCor	8 wks 4 d/wk Air bike	4 x 4 minutes at 90% of HR_max_interspersed by 3 minutes of active recovery at 70% of HR_max_	47 minutes at 70% of HR_max_
Oliveira et al.^27^	Brazil	Adults with Obesity	25 women 28±5 years HIIT:11 MICT:14	SphygmoCor	8 wks 3 d/wk Running	4x4 min at 85-95% of HR_max_interspersed by 3 min of active recovery at 65-75% of HR_max_	41 min at 65-75% of HR_max_
Toohey et al.^28^	Australia	Cancer survivors	16 adult women 51±13 years HIIT: 8 MICT: 8	SphygmoCor	12 wks 3 d/wk Treadmill running or cycle ergometer	7x30 seg at 85% of HR_max_	20 min at 55% of HR_max_

HIIT: high-intensity interval training; MICT: moderate-intensity continuous training; HR_max_: maximum heart rate; VO_2max_: maximal oxygen uptake; W_max_^:^maximum watts; d: days; wks: weeks

### Eligibility criteria

The eligibility criteria were established according to the PICOS (Population, Intervention, Comparison, Outcomes and Study design) strategy. This review included studies involving adults (18 years or older) of both genders, non-athletes and with no restrictions in terms of physical activity levels. The intervention included studies using HIIT according as defined by Weston et al.,^15^ *i.e* ., a repeated stimulus at vigorous intensity (80-100% of peak heart rate) interspersed with periods of recovery (active or passive). HIIT was compared with MICT, which consisted of a continuous stimulus at moderate intensity (54-76% of peak heart rate or equivalent). The primary outcome was cBP, measured before and after AT interventions, and the secondary outcomes were BP, arterial stiffness, and cardiorespiratory fitness. Only randomized controlled trials were considered in this review.

### Study selection

The references were systematized with the aid of a reference management software (Mendeley^®^, Elsevier, Amsterdam, Netherlands). Two researchers performed the study screening independently (GHO and VHSM). Studies whose scopes were undoubtedly out of the aim of this study and duplicate data were initially excluded from the screening process. The remaining references were assessed by title and abstract, and those who were still considered eligible underwent a full-text assessment. In case of disagreement between the two authors, they tried to reach consensus by explaining their point of view. If the disagreement persisted, a third author’s opinion (JCL) was requested for a final decision.

Exclusion criteria included (1) duplicated articles; (2) conference abstract and articles; (3) outcome measures without cBP; (4) acute design study; (5) other exercise or diet intervention associated with HIIT or MICT; (6) incomplete reports of study data.

### Data extraction

The extraction of qualitative and quantitative data was carried out independently by the two researchers (GHO and VHSM), and the data obtained were compared to avoid extraction errors. Data was extracted using a standardized spreadsheet. The demographic variables extracted from each study were: country; sample characteristics; number of subjects/age; hemodynamic assessment technique; length/frequency/mode and exercise protocols.^21^

### Risk of bias assessment

The Cochrane^®^risk of bias tool (Cochrane collaboration, Oxford, UK) was used to check the risk of bias from the included studies.^22^This tool is composed of five domains, which altogether address the methodological aspects that can influence the results of a trial. Each of the five domains have specific questions that allow five possible answers (“yes”, “probably yes”, “no”, “probably no”, and “no information”). Based on this, each domain was classified into “low”, “unclear”, or “high” risk of bias, at the author’s discretion (GHO), who assessed all the studies. The main purpose of this process was to assess the rigor of the studies’ methodology and therefore, it was not used as an exclusion criterion.

### Statistical analysis

Data were manually inserted and then pooled into the meta-analysis, which was performed by the software Review Manager^®^version 5.3 (Cochrane collaboration). Data regarding cSBP, cDBP, SBP, DBP, pulse wave velocity (PWV) and maximal oxygen uptake (VO_2max_) were presented as mean difference (MD), with 95% confidence interval (95% CI). For studies that did not provide the standard deviation (SD) of changes in the variables, the conversion to SD or the imputation of SD was made by equation according to Cochrane Handbook,^22^considering the correlation coefficient based on the data presented by Oliveira et al.^23^The random effects model was used. Forest plots were created to quantify the effects of the HIIT and MICT protocols on cSBP and cDBP. Sensitivity analysis was conducted to examine the magnitude of the influence of each study on the outcomes. A significance level of p≤ 0.05 was adopted. The heterogeneity of the studies was assessed using the I2.^22^

## Results

### Literature search

The initial searches retrieved 6115 references. After the removal of duplicates, 3677 references remained for posterior analysis of title. Subsequently, 3457 references were removed because they did not fit the PICOS questions. After reading the abstracts, 171 references were removed for not meeting the inclusion criteria. Of the 49 references selected to full-text assessment, 43 references were excluded. Finally, six studies matched the eligibility criteria and were included in the present systematic review and meta-analysis ( Figure 1 ).

**Figure 1:** – PRISMA flowchart for study selection for the systematic review and meta-analysis.

### Study characteristics

The included studies were published between 2016 and 2020 and conducted in Australia,^24 , 25^Switzerland,^26 , 27^USA^28^and Brazil.^23^The studies were conducted with sample ranging from 16 to 35 subjects, with a total of 163 participants (70% women). Most of the studies were conducted with young adults. Regarding the populations investigated in the studies, they included overweight and obese men,^24^obese women,^23^individuals with migraine,^26^depression,^27^sedentary older adults^28^and cancer survivors.^28^Of note, the assessment of cBP and arterial stiffness did not use an unique standardized method nor technique, in which the SphygmoCor^®^(AtCor Medical, Sidney, Australia)^23 - 25 , 28^and the Mobil-o-graph^®^(IEM GmbH, Stolberg, Germany)^26 , 27^were used.

In relation to cBP, one study has reported significant decreases in cSBP and cDBP following both HIIT and MICT.^25^Moreover, significant reductions in cSBP following HIIT and cDBP after MICT have been reported in two studies.^23 , 24^The other studies have not reported significant differences after the AT protocols. Concerning changes in PWV, one study found a significant reduction following HIIT and MICT,^26 - 28^wherein two studies have reported a reduction in PWV after MICT.^25 , 28^One study has verified an increase in PWV after HIIT.^25^The other three studies have not shown significant changes in this variable.^24 , 26 , 27^

### Description of the included studies

The AT programs consisted of cycling on a cycle ergometer^24 , 27^or air bike,^26^and running^23 , 26^on a running track or treadmill. One study used both cycling and running.^25^Concerning the HIIT programs, three studies^23 , 27 , 28^used the traditional Norwegian protocol,^26^which consisted of four high-intensity bouts of four minutes each. Two studies^25 , 27^used a HIIT protocol with 30-second high-intensity bouts, varying from seven to 25 stimulus. One study^24^used a HIIT protocol consisting of 10 high-intensity bouts of one minute each. The HIIT protocols had an intensity between 85% and 95% of maximum heart rate (HRmax). The MICT protocols varied from 20 to 47 minutes, in which the average duration of the MICT programs was 35.4±11 minutes, and an intensity that ranged from 55% to 75% of HRmax. Interventions lasted between four and 12 weeks with a frequency of two to four times a week. The general characteristics of the included studies are presented in Table 1 .

### Risk of bias assessment

Assessment of the risk of bias of the included studies showed an overall low risk of bias ( Figure 2 ). Only two studies showed a high risk of bias (33,3%); this indicates that the studies rigorously followed the methodological procedures proposed, which demonstrate a good methodological quality. Few issues mostly related to allocation concealment and blinding of participants were observed in some studies. Nevertheless, these issues are typical limitations in exercise intervention studies, and they do not necessarily represent poor methodological quality. Furthermore, the limitations verified were properly mentioned in the limitations section of the respective studies. It is important to emphasize that this assessment was not performed as an exclusion criterion and was used for informational purposes only. The positive and negative signs, and question marks represent low, high and uncertain risk of bias, respectively. Yet, the leave-one-out sensitivity analysis showed that the results of this meta-analysis were not driven by any particular study.

**Figure 2:** – Cochrane risk of bias assessment from the included studies.

### Synthesis of results

The HIIT was superior to MICT in reducing cSBP (MD = -3.12 mmHg, 95% CI: -4.75 to -1.50, p = 0.0002). Comparisons between baseline and post-exercise program values showed that HIIT was able to reduce cSBP significantly (MD = -3.08 mmHg, 95% CI: -5.36 to -0.81, p = 0.008); in contrast, no significant differences were observed for MICT (MD = 0.02 mmHg, 95% CI: -1.62 to 1.66, p = 0.98) ( Figure 3 ).

**Figure 3:** – Forest plot of the between-group comparison of the effects of high-intensity interval training (HIIT) versus moderate-intensity continuous training (MICT) on central systolic blood pressure: (a) HIIT pos versus pre; (b) MCIT pos versus pre; and (c) HIIT versus MICT.

The pooled analysis showed no significant differences between HIIT and MICT regarding changes in cDBP (MD = 0.08 mmHg, 95% CI: -0.97 to 1.12, p = 0.89). The same was verified for HIIT (MD = -0.36 mmHg, 95% CI: -1.49 to 0.77 mmHg, p = 0.54) and MICT (MD = -1.34 mmHg, 95% CI: -2.82 to 0.15, p = 0.08) in comparison to their respective baseline values ( Figure 4 ).

**Figure 4:** – Forest plot of the between-group comparison of the effects of high-intensity interval training (HIIT) versus moderate-intensity continuous training (MICT) on central diastolic blood pressure: (a) HIIT post versus pre; (b) MICT post versus pre; and (c) HIIT versus MICT.

Concerning the secondary variables, the pooled analysis demonstrated that HIIT was superior to MICT in reducing SBP (MD = -2.67 mmHg, 95% CI: -5.18 to -0.16 mmHg, p = 0.04). In addition, HIIT was also superior in increasing the VO_2max_(MD = 2.49 mL/kg/min, 95% CI: 1.25 to 3.73, p = 0.001). However, the pooled analysis did not show significant differences between HIIT and MICT for DBP (MD = 0.06 mmHg, 95% CI: -1.36 to 1.48], p = 0.94) and PWV (MD = -0.07 m/s, 95% CI: -1.81 to 1.68, p = 0.94) ( Table 2 ).

**Table 2:** – Mean difference and standard mean deviation comparison between HIIT and MICT on blood pressure parameters, arterial stiffness and cardiorespiratory fitness

Variable	References	N	DM	ICI	SCI	p	I^2^	P
**SBP**	23,24,25,26,27,28	154	-2.67	-5.18	-0.16	0.04	0%	0.91
**DBP**	23,24,25,26,27	139	0.06	-1.36	1.48	0.94	0%	0.98
**PWV**	23,24,25,26,27,28	153	-0.07	-1.81	1.68	0.94	0%	0.85
**VO_**2max**_**	24,25,26,27	111	2.49	1.25	3.73	0.001	0%	0.82

SBP: systolic blood pressure; DBP: diastolic blood pressure; PWV: pulse wave velocity; VO_2max_: maximal oxygen uptake; MD: mean difference; ICI: inferior confidence interval; SCI: superior confidence interval; p: p value for group comparison; I^2^: heterogeneity; P: p-value for heterogeneity; N: number of participants.

## Discussion

This is the first study to systematize and compare the effects of HIIT versus MICT on cBP in healthy and chronically diseased individuals. The main finding of this meta-analysis was that HIIT was superior to MICT in reducing cSBP (MD = -3.12 mmHg, 95% CI: -4.75 to -1.50, p = 0.0002. Previous meta-analyses have shown that HIIT and MICT are equally effective in improving ambulatory BP in pre-hypertensive^17^, and hypertensive individuals.^17 , 18^HIIT and MICT have promoted a reduction of 5.6 and 3.7 mmHg in pSBP and 4.8 and 2.4 mmHg for DBP in hypertension individuals, respectively.^18^Our findings add to the existing knowledge by demonstrating that HIIT appears to be superior to MICT in reducing cSBP, with a reduction of -3,2 mmHg. Nevertheless, there were no statistical differences between HIIT and MICT as regards changes in cDBP.

Although pBP is widely used in clinical practice, consistent evidence has been suggesting that cBP is a superior independent predictor of organ damage and cardiovascular mortality than pBP.^4 , 5 , 7^However, despite evidence supporting the prognostic importance of cBP and its distinct response to antihypertensive drug treatments when compared to pBP, not much is known about the impact of exercise training on cBP. A recent meta-analysis conducted by Zhang et al.^12^have found a reduction of approximately 6 mmHg in cSBP following AT. On the other hand, Evans et al.^29^have not found a significant reduction in cSBP (MD = -3.58 mmHg, 95% CI: -8.17 to 1.01, p = 0.13) after resistance training alone or combined with AT. The present meta-analysis demonstrates that HIIT is superior to MICT in reducing cSBP, but no difference was observed for cDBP. Therefore, aside from the distinct effects that some antihypertensive drugs have on BP depending on the analyzed site, the type of AT can also exert different effects on BP.

The mechanisms by HIIT could reduce cBP are still uncertain. While HIIT appears to be similar to MICT in reducing arterial stiffness,^19^it has been shown to be superior to MICT in improving endothelial function.^14 , 30^This improvement may contribute to the reduction of peripheral vascular resistance, which may attenuate the impedance mismatches between the central and peripheral vessels, decreasing the velocity of reflected wave to the aorta and consequently less amplification of cSBP.

In the second analysis, we found a superiority of HIIT compared to MICT in reducing SBP (MD = -2.67 mmHg, 95% CI: -5.18 to -0.16, p = 0.04). This finding diverges from other meta-analyses which found no differences in SBP between the AT modalities in pre- and hypertensive patients.^17 , 18^This discrepancy may be related to the absence of individuals with hypertension in our study, which may have produced a distinct response to AT, particularly after HIIT. Considering the pressure wave amplification phenomenon,^8 , 31^it was expected that significant changes in cBP promoted by HIIT would be transferred to the periphery, resulting in a reduction in pBP. Additionally, HIIT was also superior in increasing cardiorespiratory fitness, corroborating with previous studies that compared these AT modalities on VO_2max_in different populations.^14 , 15 , 30^On the other hand, we found no significant differences between HIIT and MICT for PWV (MD = -0.07 m/s, 95% CI: -1.81 to 1.68, p = 0.94). These findings are in line with those from Way et al.,^15^who did not observe significant differences between HIIT and MICT for PWV (MD = 0.004 m/s, 95% CI: -0.25 to -0.26 m/s, p = 0.97). The main results are illustrated in the central illustration .

**Central Illustration:** : Effects of High-Intensity Interval Training on Central Blood Pressure: A Systematic Review and Meta-Analysis

Main results of the study.

This study has some worth mentioning limitations. Firstly, there is a clear paucity of studies analyzing cBP, even if it is recognized as a strong and clinically relevant indicator of cardiovascular risk. Since this measurement has not been commonly used so far, we had to combine studies with different populations and clinical conditions in the pooled analysis. Moreover, the different methods for AT prescription, in addition to different equipment used for cBP and arterial stiffness can influence the analyses. Also, despite being validated, the methods used to evaluate cBP use indirect methods through oscillometry, which should be interpreted with caution. Another limitation which is worth mentioning is the fact that the assessment of the risk of bias was conducted by one researcher only. Lastly, although we have rigorously followed the PRISMA guidelines, some potential references might have been erroneously missed out during the screening process.

## Conclusion

In summary, HIIT was superior to MICT in reducing cSBP, but not different from MICT regarding effects on cDBP or PWV. This is a relevant finding considering that cBP is a strong and clinically predictor of cardiovascular events Future studies are required to compare the effects of HIIT and MICT on cBP in specific populations, such as prehypertensive and hypertensive individuals, who are more exposed to impairments in hemodynamic parameters.

## * Supplemental Materials

For suppementary table 1 please click here .

For suppementary table 2 please click here .
